# Rapid development of cyanobacterial crust in the field for combating desertification

**DOI:** 10.1371/journal.pone.0179903

**Published:** 2017-06-23

**Authors:** Chan-Ho Park, Xin Rong Li, Yang Zhao, Rong Liang Jia, Jae-Seoun Hur

**Affiliations:** 1 Korean Lichen Research Institute, Sunchon National University, 255 Jungang-Ro, Suncheon, Korea; 2 Cold and Arid Regions Environmental & Engineering Research Institute, Chinese Academy of Sciences, Lanzhou, China; University of Milan, ITALY

## Abstract

Desertification is currently a major concern, and vast regions have already been devastated in the arid zones of many countries. Combined application of cyanobacteria with soil fixing chemicals is a novel method of restoring desertified areas. Three cyanobacteria, *Nostoc* sp. Vaucher ex Bornet & Flahault, *Phormidium* sp. Kützing ex Gomont and *Scytonema arcangeli* Bornet ex Flahault were isolated and tested in this study. Tacki-Spray^TM^ (TKS7), which consists of bio-polysaccharides and tackifiers, was used as a soil fixing agent. In addition, superabsorbent polymer (SAP) was applied to the soil as a water-holding material and nutrient supplement. Application of cyanobacteria with superabsorbent polymer and TKS7 (CST) remarkably improved macro-aggregate stability against water and erodibility against wind after 12 months of inoculation when compared to the control soil. The mean weight diameter and threshold friction velocity of the CST treated soil were found to be 75% and 88% of those of the approximately 20-year-old natural cyanobacterial crust (N-BSC), respectively, while these values were 68% and 73% of those of the N-BSC soil after a single treatment of cyanobacteria alone (CY). Interestingly, biological activities of CST were similar to those of CY. Total carbohydrate contents, cyanobacterial biomass, microbial biomass, soil respiration, carbon fixation and effective quantum yield of CST treated soil were enhanced by 50–100% of the N-BSC, while those of control soil were negligible. Our results suggest that combined application of cyanobacteria with soil fixing chemicals can rapidly develop cyanobacterial crust formation in the field within 12 months. The physical properties and biological activities of the inoculated cyanobacterial crust were stable during the study period. The novel method presented herein serves as another approach for combating desertification in arid regions.

## Introduction

Desertification is a global environmental issue in which land in arid, semiarid and dry sub-humid areas is degraded by factors such as climatic variations and anthropogenic activities [1]. Problems associated with desertification include loss of biodiversity, low soil productivity, dust storms and economic losses. Drylands cover 41.3% of all land and are home to more than 2.5 billion people worldwide [2, 3]. Desertification has reportedly affected 10 to 20% of these lands, on which 250 million people live, while another 1 billion people live in areas at risk of undergoing further desertification [2, 4]. Therefore, combating desertification is a major challenge facing humanity.

Various treatments have been employed to stabilize desertified soil. Several chemicals such as organic polymers have been investigated for their ability to stabilize sand, which is essential to increasing sand aggregate stability and protecting sand particles against wind erosion. Investigation of the chemicals, PASP (poly aspartic acid), PVA (polyvinyl alcohol), PAM (polyacrylamide) and Gypsum [5–9] have revealed that they are less expensive than mechanical and vegetative materials and are able to effectively stabilize soil particles [10]. However, this method is not considered to be an ecological restoration method [5, 11]. A common and widely adapted method of stabilizing desertified soil is planting native vegetation. However, monotonous cultivation of the same species of trees is susceptible to widespread damage caused by diseases or pests. Moreover, the annual precipitation of arid regions is generally less than 300 mm, while the evaporation of trees is over 3000 mm [12]. Hence, plants may not survive without proper management of water supply, and may even drain groundwater from nearby grasslands.

Cyanobacterial crusts, which are commonly found in arid and semiarid regions, are dominated by cyanobacteria, but can also contain green micro-algae, bacteria and micro-fungi [13]. Cyanobacterial crusts have been considered one of the solutions for the restoration of degraded soil. Morphologically tangled filamentous cyanobacteria improve soil aggregate stability by adding organic matter and secreting extracellular polymeric substances (EPSs) [14–18]. Cyanobacteria can also survive extreme environmental conditions such as high or low temperature, acidic or alkaline environments, and salinity, low precipitation, strong irradiation, and desiccation. Furthermore, cyanobacteria improve soil fertility through mineral chelation, dust entrapment, and nutrient fixation, which are beneficial to plants and animals [19, 20]. However, the recovery times are required for cyanobacterial crusts are predicted to be several decades under natural environmental conditions [21]. Therefore, methods enabling rapid development of inoculated cyanobacterial crusts within a limited time span are being considered. Many studies have also reported the feasibility of inoculating cyanobacterial cells to induce rapid development of BSCs to improve soil stability, productivity and nutrition [22–29].

Many factors destroy early stage cyanobacterial crusts under natural environmental conditions. Raindrops destroy soil aggregates, leading to the detachment of soil particles from cyanobacterial crusts [30]. Additionally, grazing and trampling of livestock cause decreases or loss of biological soil crusts (BSCs) [31–33]. Moreover, frequent disturbances keep BSCs in the early successional stage for long periods of time, or induce regression from late successional BSCs to the early stage [34]. Combined application of cyanobacteria with soil fixing chemicals has the potential to stabilize the early stages of inoculated cyanobacterial crust and accelerate progression to the next successional stage. Non-toxic, eco-friendly materials suitable for soil stabilization have been considered for use with cyanobacteria. Tackifiers based on naturally occurring resins are used to increase the stickiness of adhesives. TKS7 (Tacki-Spray^TM^) is a commercially available product made from tackifiers and bio-polysaccharides extracted from plant seeds. Bio-polysaccharides, the main components of TKS7, are expected to enhance soil aggregation, playing a role similar to that of EPS secreted from cyanobacteria in the soil. Moreover, superabsorbent polymer (SAP) can be applied to soil as a water-holding material and nutrient supplement [35]. The feasibility of combined application of soil fixing chemicals with cyanobacteria was described for the rapid development of inoculated cyanobacterial crust under laboratory conditions [36]. In the present study, we conducted field experiments to evaluate the feasibility of combined application of soil fixing chemicals with cyanobacteria under natural conditions.

## Materials and methods

### Mass cultivation of cyanobacteria

**Fig 1:**
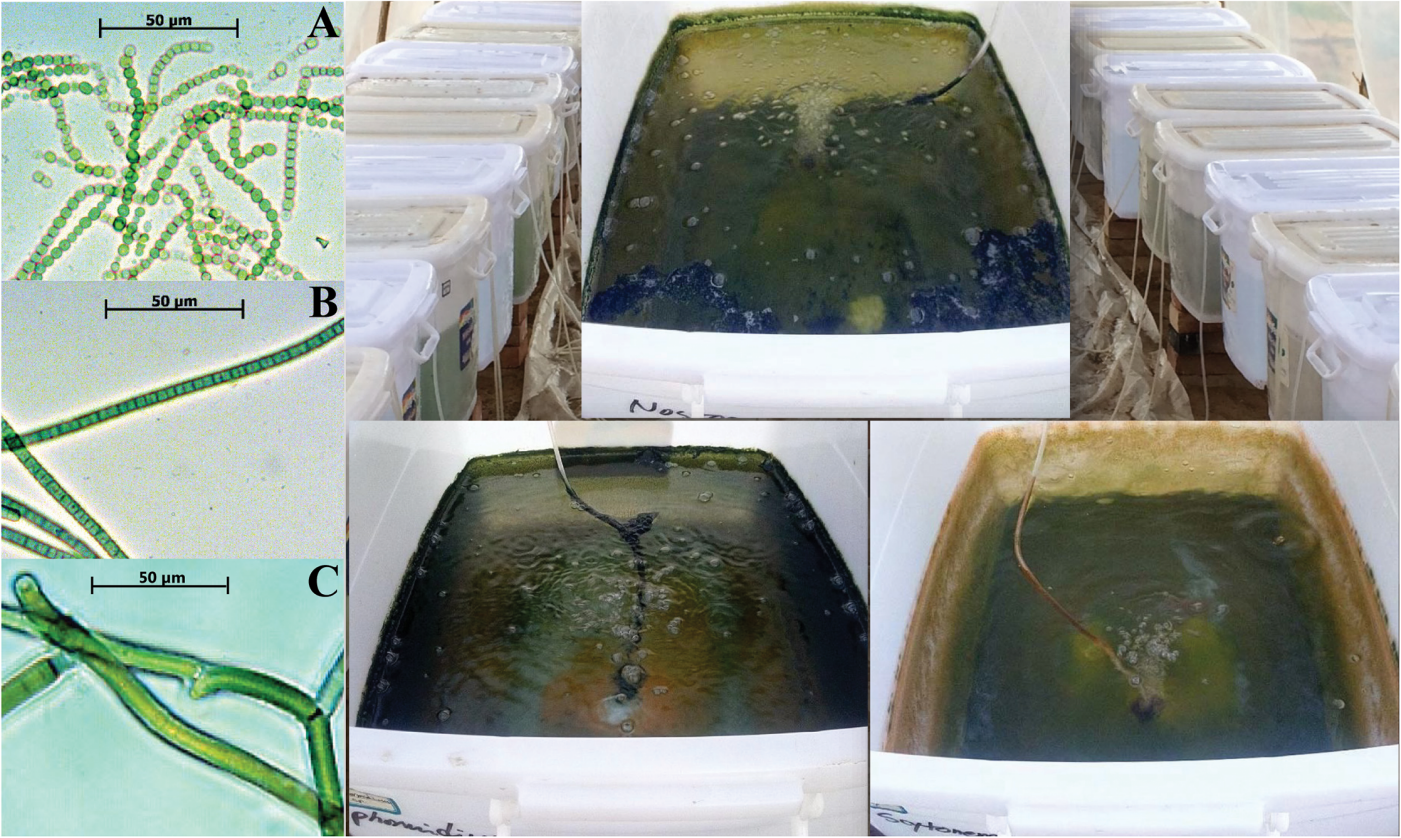
Light microscopy images of *Nostoc* sp. (A), *Phormidium* sp. (B) and *Scytonema arcangeli* (C) and their inoculum in plastic containers.

Sampling permissions were granted by the Cold and Arid Regions Environmental & Engineering Research Institute, Chinese Academy of Sciences. The field studies did not involve endangered or protected species. Soil samples for isolating cyanobacteria were collected from the Tengger Desert in Ningxia Province, Northern China (37°25’33.2“N, 104°40’20.5“E). Cyanobacterial isolates were determined and cultured as previously described [36]. A cyanobacterial consortium consisting of *Nostoc* sp. Vaucher ex Bornet & Flahault, *Phormidium* sp. Kützing ex Gomont and *Scytonema arcangeli* Bornet ex Flahault was used (Fig 1). Cyanobacteria were cultured in mass in a translucent plastic container (53×40×30 cm) filled with BG-11 liquid medium [37] for 2 weeks from 24 May to 22 August 2014 (Fig 1). All equipment for mass cultivation was set up in a greenhouse and culturing containers were covered with a lid until cell harvest. The top of the greenhouse was covered with a black net to provide reasonable light intensity and temperature. The mean temperature of the liquid medium was 23°C under a light intensity of 400 μmol photons m^-2^ s^-1^. Cyanobacterial cells were harvested during the exponential growth phase by centrifugation at 1400 g for 5 min. Cyanobacteria cultured in the laboratory were used as a seed for mass cultivation in the field at Shapotou Desert Research and Experiment State Key Station, Ningxia Province, Northern China.

### Field experiment design

Field experiments were conducted at the Shapotou Desert Research and Experiment State Key Station, Ningxia Province, Northern China (37°27’36.8“N, 105°00’42.7“E). The annual precipitation in this area is less than 300 mm [38], and rainfall occurs from June to September. The surface soil crust was completely removed from each experimental plot (4 m^2^) to generate equal conditions, after which plots were covered with bare sand collected from a depth of 30 centimeters. The following three treatments were used for the field experiment: 1) control soil (Con); 2) cyanobacteria application (CY); 3) combined application of cyanobacteria with SAP and TKS7 (CST). Cyanobacteria were applied at 200 g fresh weight (FW) m^-2^ (10 g dry weight (DW)) by spraying three times from June to August 2014 for CY. At the same time, SAP and TKS7 were applied at a concentration of 10 and 1 g DW m^-2^, respectively, together with cyanobacteria during the first application, while the other two applications were only sprayed with cyanobacteria for CST. TKS7 (Tacki-Spray^TM^) was purchased from STBi CO., Hwasung, Korea and superabsorbent polymer (SAP, WCS-0907) was purchased from Greenfield Co., Ltd., Korea. The same amount of water was applied to the control plot instead of the treatment. Next, 5 mm of water per square meter was sprayed onto the plots once a day for 1 week. For comparison, natural cyanobacterial crust (N-BSC) was also analyzed. N-BSC was classified as an algae-dominated crust approximately 20 years old according to Liu *et al*. [38].

### Field soil sampling

Polystyrene Petri-dishes (50×10 mm) were used for regular sampling of the treated soils. The Petri-dishes were turned over and gently pressed into the soil until the bottom reached the same level of the soil surface. Soil samples were collected from depths of 10 mm at 10 random points in each treatment plot during October of 2014 and July of 2015 after 4 and 12 months of development in the field, respectively. Four collected samples were used for the aggregate stability test, three samples were used to determine the fluorescence yield, and another three samples were used for analysis of the chlorophyll *a*, total carbohydrate contents, organic carbon and microbial carbon. For chemical analysis, soil samples were air dried and leaves and twigs were removed, after which soils were partially ground.

### Aggregate stability

We conducted a fast wetting test for soil aggregate stability as described by Park *et al*. [36]. Fast wetting is useful for testing the behavior of dry soils under fast wetting events such as heavy rains during summer. The MWD (Mean Weight Diameter) is the total of the mass fraction of soil left on each sieve after wetting.

### Threshold friction velocity (TFV)

**Fig 2:**
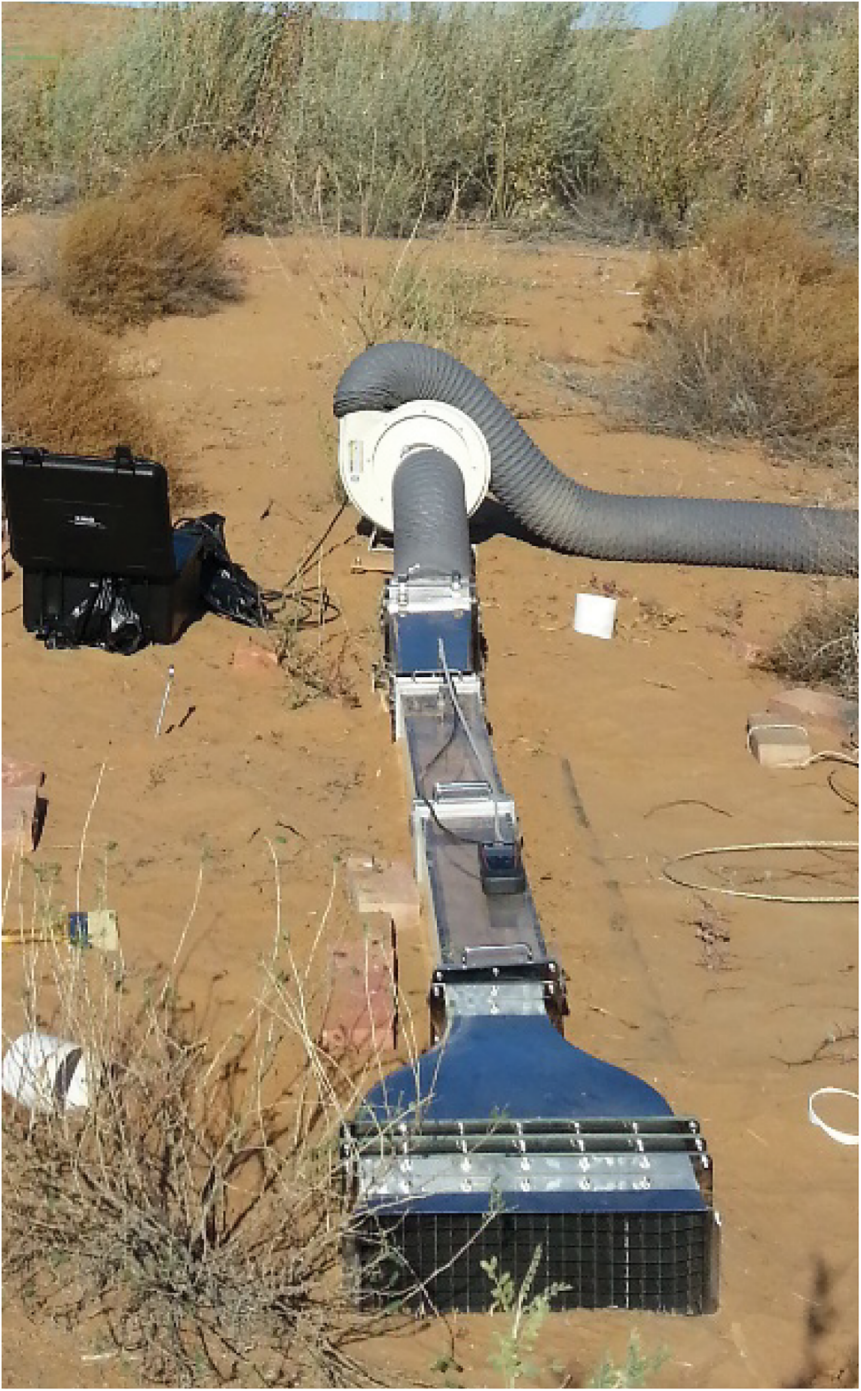
Wind tunnel test in the field.

The threshold friction velocity (TFV) was determined using a potable wind tunnel that was designed following the specifications described by Belnap *et al*. [39]. The tunnel is open-bottomed, with a 150×150 mm cross section, a 2.4 m length of transparent polycarbonate and a 3:1 contraction section with a honeycomb flow straightener (Fig 2). Wind speed was estimated by a pitot tube (Ø3 mm) inside the tunnel attached to a MP120 manometer (KIMO, Montpon, France). Wind tunnels were placed on each experiment plot and the pitot tube was set at 75 mm above the soil surface. Wind speeds were then gradually increased until the threshold friction velocity of each cyanobacterial crust was reached, after which they were maintained for 3 min. The threshold friction velocity was defined as the value at which particles or small fragments were initially detached and moved forward from the soil surface [40]. Wind velocity was then recorded when the soil particles were removed from the crust surface.

### Cyanobacterial biomass

The biomass of cyanobacteria was determined by measuring the chlorophyll *a* concentration. Briefly, 2 g DW of soil were added to 5 mL of ethanol (99.9%) in a 50 mL cap tube, then placed into an 80°C water bath for 5 min. Next, samples were allowed to cool for 30 min, after which they were centrifuged at 2500 g for 5 min. The absorbance of the supernatant was subsequently measured (A665) and the chlorophyll *a* concentration was estimated using a previously described equation [41]. All treatments were conducted in triplicate.

### Total carbohydrate contents

The soil total carbohydrate contents were determined according to the method described by Safařík and Šantrůčková [42]. Briefly, 5 mg DW of soil aggregates were placed into a test tube (100×12 mm), after which 1 mL of distilled water and 5% phenol solution were added and vortexed. Next, 5 mL of concentrated sulfuric acid were added and the solutions were vortexed for 10 sec, then incubated at room temperature for 1 h. Blanks were subsequently prepared with distilled water instead of phenol solution. The tubes were then centrifuged at 3300 g for 10 minutes, after which the absorbance of the supernatant was measured at 485 nm. A linear regression curve was obtained using glucose (Sigma-Aldrich, St. Louis, MO, USA) as a standard polysaccharide material, after which the polysaccharide concentration was calculated using a regression equation. All treatments were conducted in triplicate.

### Organic carbon

Soil organic C was measured according to the WLOI (weight loss on ignition) method described by Wang *et al*. [43]. Briefly, approximately 3 g of soil were placed in a crucible and dried at 105°C for 12 h to determine the DW of the samples, after which they were put in a muffle furnace (Hy-4500S, YUYU Scientific, Ansan, Korea) and combusted for 12 h at 500°C. The samples were then cooled to room temperature in a desiccator and weighed. All treatments were conducted in triplicate.

### Microbial biomass carbon

Soil microbial biomass was determined according to the chloroform fumigation and extraction method described by Hobbie [44]. For non-fumigated samples, 10 g DW of soils were immediately extracted with 50 mL of 0.5 M K_2_SO_4_, then placed on a shaker for 1 h. After shaking, extracted solutions were filtered through Whatman No. 1 filter paper and stored in the freezer. For fumigated samples, beakers with 10 g DW of soil were placed into a vacuum desiccator together with a 50 mL beaker containing boiling chips and 20 mL of chloroform in the middle of the desiccator. The desiccator was then evacuated until the chloroform boiled, after which it was vented four times repeatedly. The samples were subsequently incubated in the dark for 3 days at 25°C. Following incubation, the vacuum was released and excess chloroform removed. Next, the chloroform fumigated samples were extracted with 50 mL of 0.5 M K_2_SO_4_, then placed on a shaker for 1 h. After shaking, extracted solution was passed through Whatman No. 1 filter paper and stored in the freezer. Total dissolved carbon was determined using a TOC analyzer (GE Sievers Innovox TOC, Boulder, CO, USA). All treatments were conducted in triplicate.

$$C_{mic}=E_{c}/K_{c}$$

Where, *C*_*mic*_ = *microbial biomass carbon*,
*E*_*c*_ = *fumigated carbon* – *non-fumigated carbon* and
*K*_*c*_ = 0.45

### Soil respiration

Soil CO_2_ release was measured in October, 2014 and July, 2015 after 4 and 12 months of development in the field using a Li-6400-09 Soil Chamber (LI-COR, Lincoln, USA). PVC collars (10.4 cm in diameter, 12 cm in height, inserted ~10 cm) were installed in the experimental plots in August of 2014. The soil surfaces were gently moistened with 5 mm of water 1 h before the measurements, which were conducted from 10:00 am to 10:30 am (GMT +8). For each measurement, CO_2_ release was recorded at 4 s intervals over a 40 s period once steady state conditions were achieved within the chamber. All treatments were conducted in quadruplicate.

## Carbon fixation

Carbon fixation was measured on a clear day using Licor 6400 portable photosynthesis systems (LI-COR Inc. Lincoln, NE, USA) with a 137 cm^2^ transparent polycarbonate chamber that fit over the soil collar. The soil surfaces were gently moistened with 5 mm of water 2 h before the measurements and soil samples were collected with a cylinder (42×3 mm). Gross photosynthesis was measured in the light (*P*_g_), and respiration (*R*_e_) was measured in the dark by placing opaque foil over the chamber. Net photosynthesis was calculated by subtracting *R*_e_ from *P*_g_, which gave the *P*_n_ of the cyanobacterial crust. These photosynthetic rates were calculated on a surface area basis as μmol CO_2_ m^-2^ s^-1^. The *P*_n_ was calculated to determine for hourly carbon fixation (H, mg C m^-2^) using the following equation.

$$\lfloor H\rfloor =P_{n}\times (0.000012\times gC/\mu mol\;{CO}_{2})\times {3600s∙h}^{-1}\times 1000$$

Where, H = *hourly carbon fixation* and
*P*_*n*_ = *net photosynthesis*

### Light response curves for quantum yield of PSII and relative electron transport rate (rETR)

The light saturation curve was measured by Pulse-Amplitude-Modulation (PAM-2500, Heinz, Walz, Germany). Soil samples were collected in experiment plots, then moved into the laboratory, and stabilized for 1 week. To determine the fluorescence yield, soil samples were moistened with 2 mm water and dark-adapted for at least 20 min before measurement. The PSII efficiency and relative Electron Transport Rate (rETR) of the soil were estimated under continuous actinic light. A PAM light probe was connected to the cuvette to expose soil to eight light steps (each 30 s) ranging from 7 to 1391 μmol photons m^−2^ s^−1^ PAR. Actinic light was provided by a red LED (630 nm). The parameters were determined based on the following formulas developed by Schreiber *et al*. [45]:

$$PSII\;efficiency\;(\Phi_{PSII})=\left(Fm^{\prime }-Fs\right)/Fm^{\prime }=\Delta F/Fm\prime$$

$$Relative\;electron\;transport\;rate\;(rETR)=\Phi_{PSII}\times PFD\times 0.5\times 0.84$$

Where, *Φ*_*PSII*_ = *effective quantum yield*,
Fs = *steady-state level fluorescence during actinic illumination*,
PFD = *irradiance* (*mol m*^−2^ *s*^−1^ *PAR*),
0.5 = *the PSI/PSII allocation factor*, and
0.84 = *the mean absorbance factor for the plant* [46]

All treatments were conducted in triplicate.

### Statistical analysis

Data were analyzed using SPSS 18.0 (SPSS, Inc, Chicago, IL, USA) and a p<0.05 was considered significant for all analyses. Differences in mean weight diameter, total carbohydrate, chlorophyll *a*, organic carbon, soil respiration, carbon fixation, microbial carbon and threshold friction velocity values among treatments were analyzed by one-way ANOVA followed by Duncan’s post hoc tests.

## Results

### Soil aggregate stability

**Fig 3:**
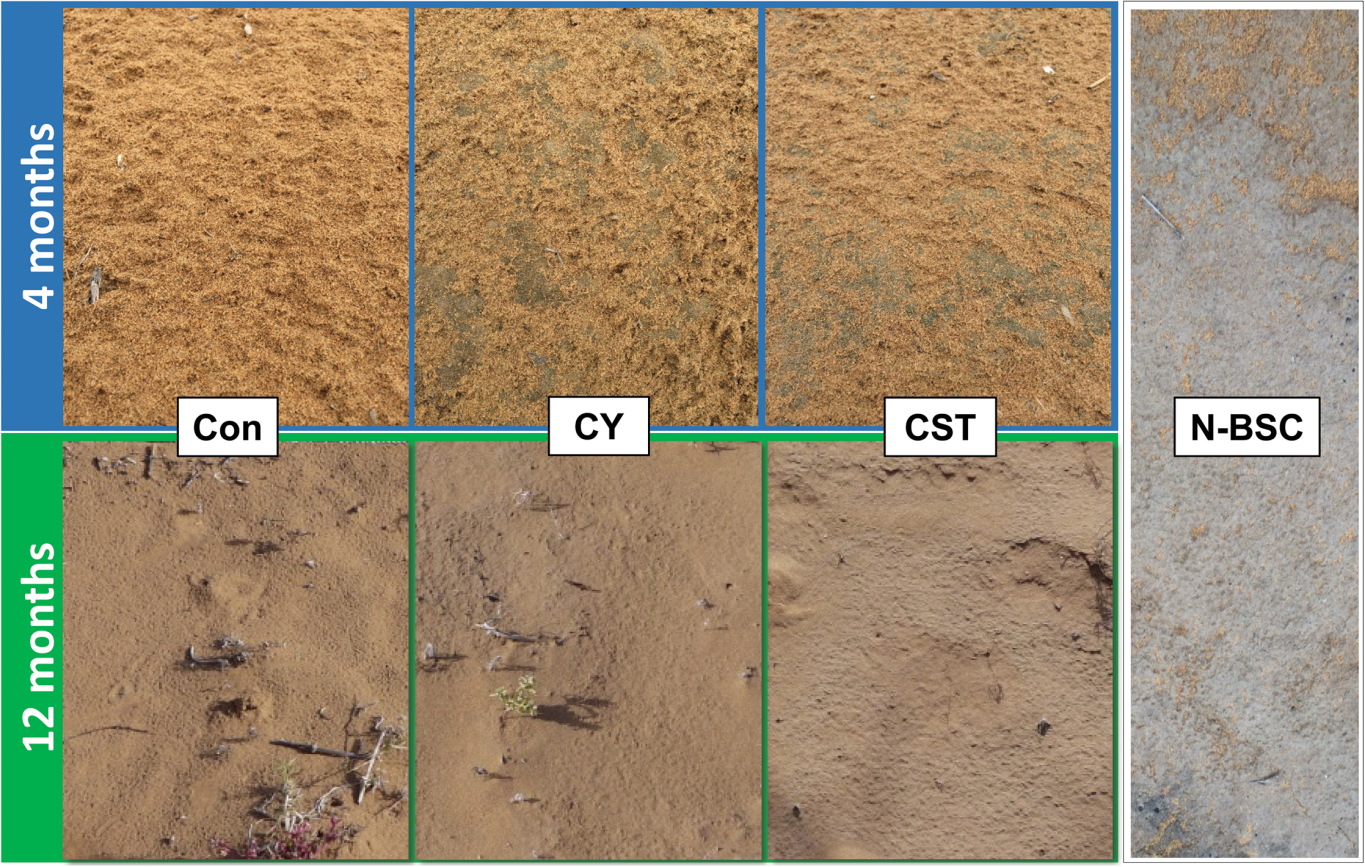
Rapidly formed cyanobacterial crust images in the field at 4 and 12 months after inoculation. (Con) control soil; (CY) cyanobacteria treated soil; (CST) cyanobacteria with SAP and TKS7 treated soil; (N-BSC) natural cyanobacterial crust. The photos of the 4 month old crusts were taken when the soil was wet. Photos of the 12 month old crusts and the N-BSC were taken when the soil was dry.

Cyanobacterial crust was formed by application of cyanobacteria alone or with soil fixing chemicals together in the field after 4 and 12 months of development (Fig 3). After development for 4 months, the crusts formed following inoculation with CY and CST appeared to be premature, with green-gray surfaces under wet conditions. Under wet conditions, CY and CST were soft, and water was quickly absorbed (within 2–3 seconds). The surface of soils that received CY and CST were evenly flat, and sand particles were firmly fixed by cyanobacteria and TKS7. The developmental states of control soil (Con), CY and CST treated soil were notably different at 12 months after inoculation. Specifically, almost no cyanobacterial crust was found in control soil; however, an aggregated soil crustal mass was observed in CY and CST soil, and the soil crust was more prominently developed in CST soil than CY soil.

**Fig 4:**
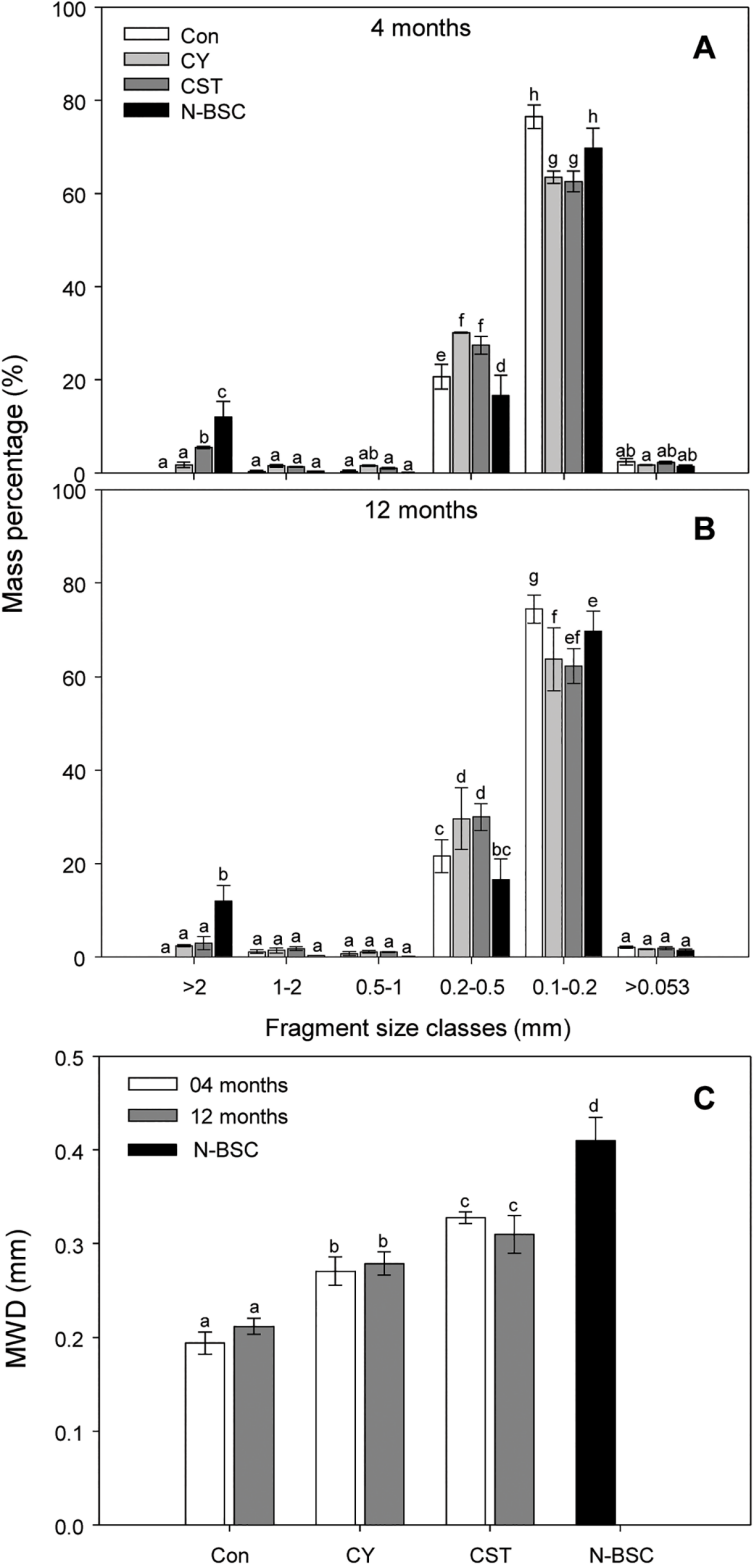
**Fragment size distribution (A, B) and mean weight diameter values (C) of cyanobacterial crusts after 4 and 12 months of development.** (Con) control soil; (CY) cyanobacteria treated soil; (CST) cyanobacteria with SAP and TKS7 treated soil; (N-BSC) natural cyanobacterial crust. Values represent the means of quadruplicate measurements. Values with the same letter are not significantly different according to the multiple comparison test (95% Duncan’s post hoc tests). Error bars represent the standard errors.

The fragment sizes of inoculated crusts were analyzed at 4 and 12 months after inoculation in the field (Fig 4A and 4B). The CST increased soil aggregate stability as indicated by the percentage of coarse fragments (>2 mm) increasing by two times relative to CY after 4 months of development. Coarse fragments of crusts formed by inoculation with CY and CST comprised up to 30% of the approximately 20-year-old natural cyanobacterial crust (N-BSC). MWD values also confirmed that the CST improved soil aggregation (Fig 4C). The MWD value of CST was 0.33 mm, while the CY was 0.27 mm after 4 months of development. In addition, the MWD values of CST and CY were 0.31 mm and 0.28 mm, respectively, after 12 months of development. The MWD value of the CST soil crust increased to more than 75% of that of the N-BSC over 12 months, while the MWD values of the CY and Con were 68% and 51% of the N-BSC, respectively (Table 1).

**Table 1:** Biological and physical measurements of cyanobacterial crusts after 4 and 12 months of development.

	Month	MWD (mm)	TFV (cm s^-1^)	TC (μg g^-1^)	Chl-*a* (μg g^-1^)	C_org_ (g kg^-1^)	CO_2_ flux (μmol m^-2^ s^-1^)	CF (mg C m^2^ h^-1^)	C_mic_ (μg C g^-1^)
Con	4	0.19±0.01a	-	176.8±3.4a	0.47±0.06a	2.4±0.74a	0.34±0.06a	-	15.7±5.3a
	12	0.21±0.01a	320±20a	276.4±47.2b	0.47±0.01a	2.8±0.40ab	0.40±0.14a	8.7±0.4a	31.4±11.7ab
CY	4	0.27±0.02b	-	676.7±53.9c	3.01±0.19b	3.3±0.35bc	0.63±0.05b	-	61.7±7.2bcd
	12	0.28±0.01b	730±10b	656.5±34.1c	3.10±0.22b	3.1±0.33abc	0.76±0.18b	28.8±1.0b	93.1±27.2d
CST	4	0.33±0.01c	-	799.3±39.5d	2.78±0.35b	3.2±0.61bc	0.69±0.07b	-	58.9±9.9bc
	12	0.31±0.01c	880±15c	669.1±68.4c	2.96±0.47b	3.8±0.28cd	0.71±0.06b	31.0±1.8c	79.6±11.5cd
N-BSC	≒20 years	0.37±0.01d	1000±20d	1259.0±21.0e	4.45±0.40c	4.4±0.57d	1.04±0.14c	38.1±1.0d	137.8±33.2e

MWD, mean weight diameter; TFV, threshold friction velocity; TC, total carbohydrate; Chl-*a*, chlorophyll *a*; C_org_, organic carbon; CO_2_ flux, soil respiration; CF, carbon fixation; C_mic_, microbial biomass carbon. Each number is the mean ± standard error. Values with the same letters within the same column are not significantly different according to the multiple comparison test (95% Duncan’s post hoc tests). The bar (-) indicates no measured data.

### Threshold friction velocity

**Fig 5:**
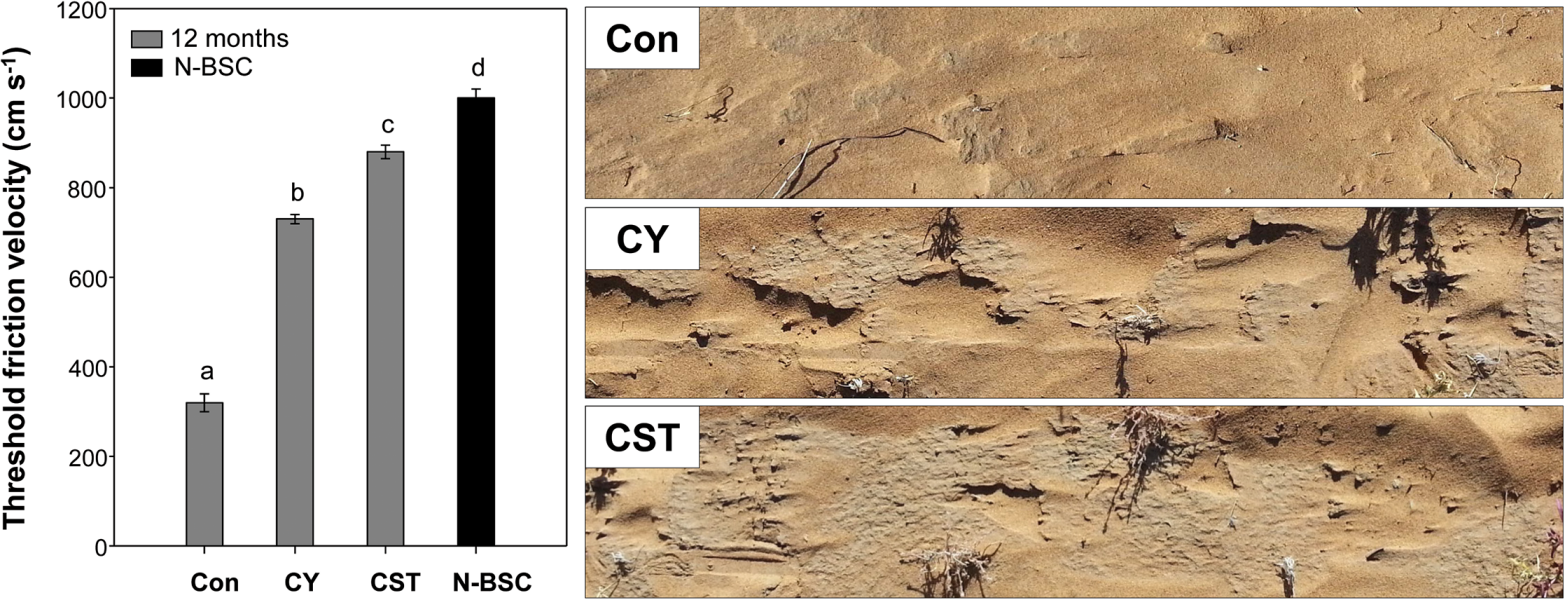
Threshold friction velocity values of cyanobacterial crusts after 12 months of development. **The images were taken after the wind tunnel test.** (Con) control soil; (CY) cyanobacteria treated soil; (CST) cyanobacteria with SAP and TKS7 treated soil; (N-BSC) natural cyanobacterial crust. Values represent the means of triplicate measurements. Values with the same letter are not significantly different according to the multiple comparison test (95% Duncan’s post hoc tests). Error bars represent the standard errors.

Wind erodibility of soil was evaluated using a portable wind tunnel. The soil fragments of cyanobacterial crust rapidly induced over 12 months were initially detached when the wind speed reached more than 880 cm s^-1^ in CST treated soil, and 730 cm s^-1^ in CY treated soil, while the control soil was 320 cm s^-1^ (Fig 5). The threshold friction velocity value of CST was significantly higher than that of CY (*p*<0.05), while it was 88% of that of N-BSC (1000 cm s^-1^). The photo images in Fig 5 were taken after the wind tunnel test. The CST plot was clearly more stable than the CY and Con plots against wind erosion, even at high wind speed (Table 1).

### Total carbohydrate contents

**Fig 6:**
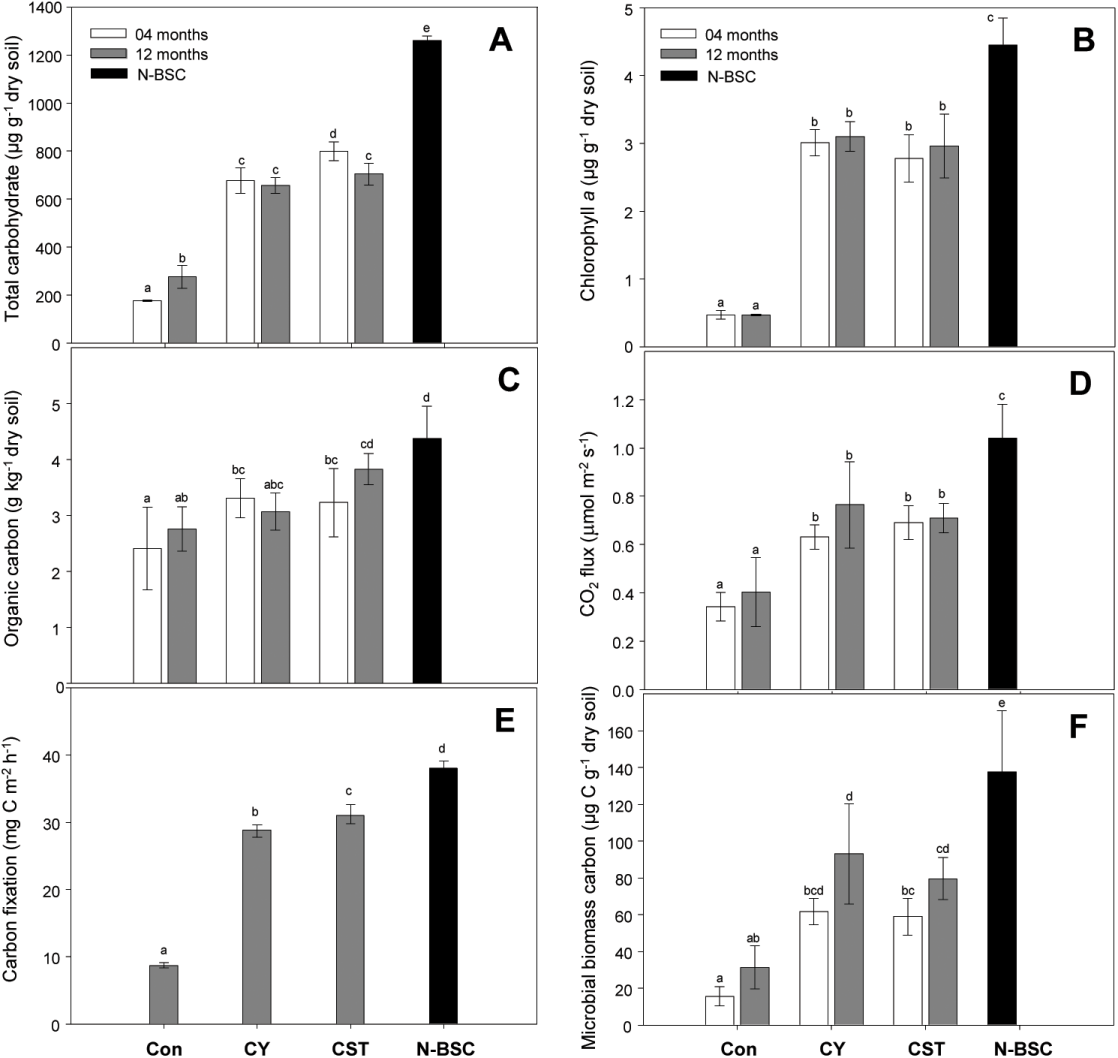
**Total carbohydrate (A), chlorophyll *a* (B), organic carbon (C), soil respiration (D), carbon fixation (E) and microbial biomass carbon (F) of cyanobacterial crusts after 4 and 12 months of development.** (Con) control soil; (CY) cyanobacteria treated soil; (CST) cyanobacteria with SAP and TKS7 treated soil; (N-BSC) natural cyanobacterial crust. Values represent the means of triplicate measurements. Values with the same letter are not significantly different according to the multiple comparison test (95% Duncan’s post hoc tests). Error bars represent the standard errors.

The total carbohydrate contents of CST, CY, Con and N-BSC were 799.3, 676.7, 176.8 and 1259 μg g^-1^, respectively, after 4 months. The total carbohydrate content of CST and CY were significantly greater than Con (*p*<0.05). The total carbohydrate content of CST was higher at 4 months than at 12 months after inoculation. This might have been due to heterotrophic organisms entering the crust community, which then consumed the total carbohydrate and hence reduced the total carbohydrate content (Fig 6A).

### Cyanobacterial biomass and soil organic carbon

Inoculation of bare sand in the field with cyanobacterial cells significantly increased cyanobacterial biomass compared to Con (based on the chlorophyll *a* concentration), and certain levels of cyanobacterial biomass were successfully maintained in the crust 4 and 12 months after inoculation. There was no significant difference in cyanobacterial biomass between CST and CY for 12 months although chemicals were included in the CST (*p*>0.05). The level of cyanobacterial biomass in the inoculated crust of CST and CY reached more than 66% (3.1–2.7 μg g^-1^) of the N-BSC (4.45 μg g^-1^) under the treatment conditions, while that of the Con was 10% (Fig 6B).

Soil organic carbon of the sandy soil in the field increased to some extent in response to inoculation with cyanobacterial cells. There was no significant difference in soil organic carbon between CST (3.2 μg g^-1^) and CY (3.3 μg g^-1^) after 4 months of inoculation (*p>*0.05). After 12 months it increased to 3.8 μg g^-1^ in CST soil and was equivalent to 86% of N-BSC (4.4 μg g^-1^), while CY soil showed no significant difference in soil organic carbon between 4 and 12 months of development (Fig 6C) (*p*>0.05).

### Soil respiration, carbon fixation and microbial biomass carbon

Inoculation of the sand with cyanobacterial cells also resulted in a significant increase in soil respiration (CO_2_ flux), carbon fixation and microbial biomass carbon relative to control soil. There was no significant difference in soil respiration between CST and CY after 4 and 12 months of induction (*p>*0.05). The CO_2_ flux values ranged from 0.63–0.76 μmol m^-2^ s^-1^, which corresponded to 68% N-BSC (1.04 μmol m^-2^ s^-1^) (Fig 6D). The hourly carbon fixation rates in inoculated cyanobacterial crusts were high, with values of 31.0 (CST) and 28.8 mg C m^-2^ h^-1^ (CY) being observed, while the rate in Con was only 8.7 mg C m^-2^ h^-1^. The hourly carbon fixation rates of CST and CY ranged from 81.4 to 75.6% of the N-BSC (38.1 mg C m^-2^ h^-1^) after 12 months of inoculation (Fig 6E). The microbial biomasses of CST (58.9 μg C g^-1^) and CY (61.7 μg C g^-1^) did not differ significantly after 4 months of development (*p>*0.05); however, after 12 months of development the microbial biomass of CST and CY increased to 79.6 and 93.1 μg C g^-1^, respectively (Fig 6F).

### Relationship of MWD to each soil property

**Fig 7:**
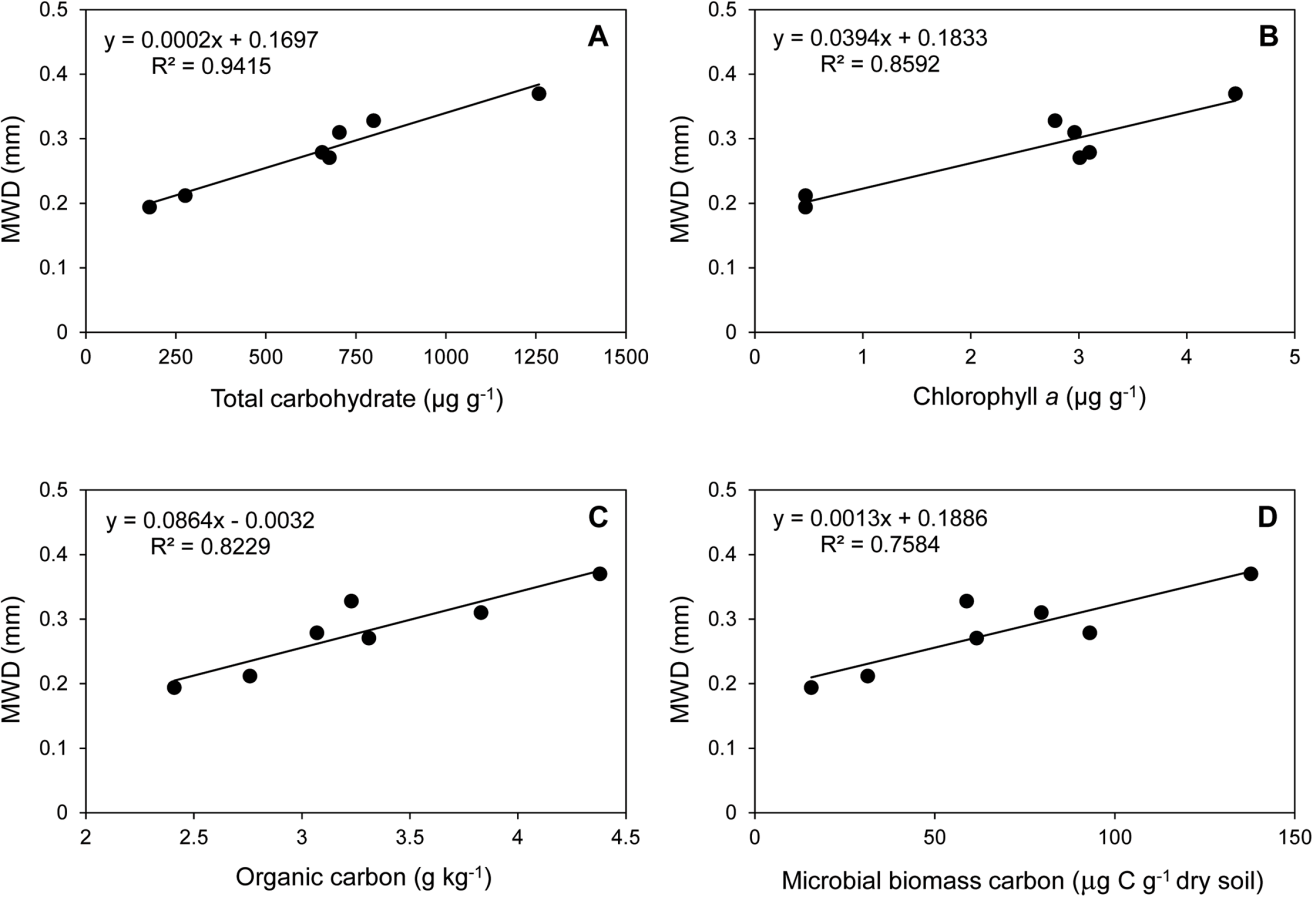
Relationship between MWD values and each soil content factor (total carbohydrate, chlorophyll *a*, organic carbon).

The total carbohydrate contents, cyanobacterial biomass, total organic carbon and microbial biomass were closely related to aggregate stability (Fig 7A–7D). The R^2^ values of the regression equation were greater than 0.75 in all factors related to the MWD values. The total carbohydrate values (0.94) were the most closely related to the MWD values, followed by the chlorophyll *a* contents (0.86).

### Light response curves for quantum yield of PSII and relative electron transport rate (rETR)

**Fig 8:**
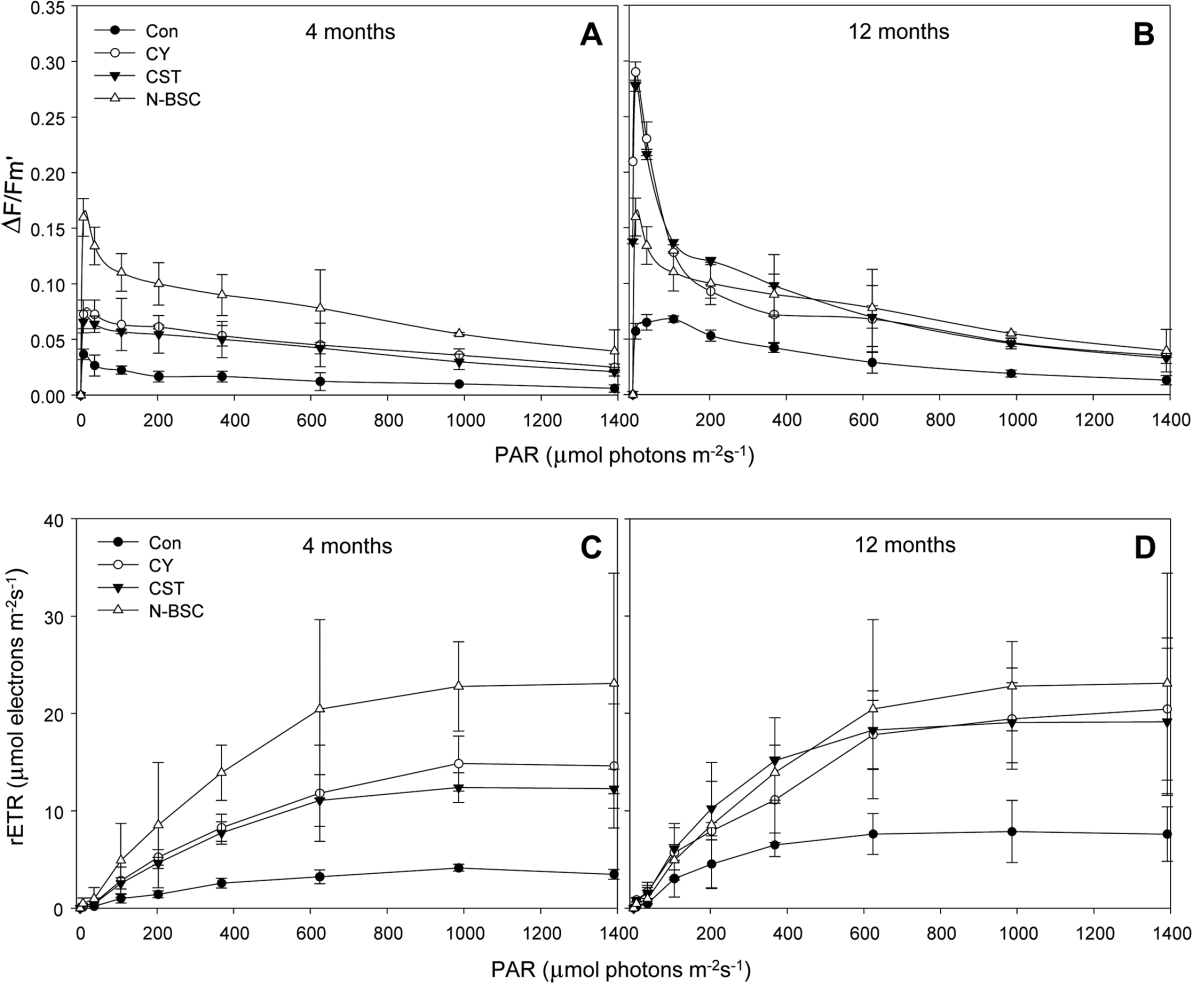
**Quantum yield efficacy of PSII (*ΔF/Fmʹ*) (A, B) and relative electron transport rate (rETR) (C, D) of cyanobacterial crust after 4 and 12 months of development.** (Con) control soil; (CY) cyanobacteria treated soil; (CST) cyanobacteria with SAP and TKS7 treated soil; (N-BSC) natural cyanobacterial crust. Values represent the means of triplicate measurements. Error bars represent the standard errors.

Cyanobacterial photosynthetic activity was evident in the inoculated crust of CY and CST, even after 4 and 12 months of development in the field. The quantum yield efficacy of PSII (*ΔF/Fmʹ*) was significantly higher in the inoculated crusts than control sand. There was no significant difference in the quantum yield of PSII between CST and CY. Although the mature natural cyanobacterial crust (N-BSC) showed the highest efficacy, the quantum yield of PSII of the inoculated crusts was more than 50% of that of the natural crust after 4 months. However, at 12 months after development the PSII values of CST and CY were greatly increased to the N-BSC level (Fig 8A and 8B). The relative electron transport rate (rETR) showed a similar response as the quantum yield of PSII. Specifically, the inoculated crusts of CST and CY showed significantly higher rETR values than the control sand, while there was no significant difference in rETR between CST and CY. The percentages were more than 50% of the N-BSC during the first 4 months of development, then increased to 100% within 12 months (Fig 8C and 8D).

## Discussion

The inoculated cyanobacterial crusts of CY and CST were successfully developed within 12 months. When visually compared to approximately 20-year-old natural cyanobacterial crust (N-BSC), the inoculated cyanobacterial crusts appeared to be in the early stage. Specifically, N-BSC was characterized by a black surface 7 mm thick, which corresponded to algae-dominated crust according to the Chinese classification [38]. However, soil physical properties and biological activities of the inoculated cyanobacterial crusts were significantly improved compared to those of non-inoculated sand (*p*<0.05), reaching a comparable level of mature natural crust within 12 months.

**Fig 9:**
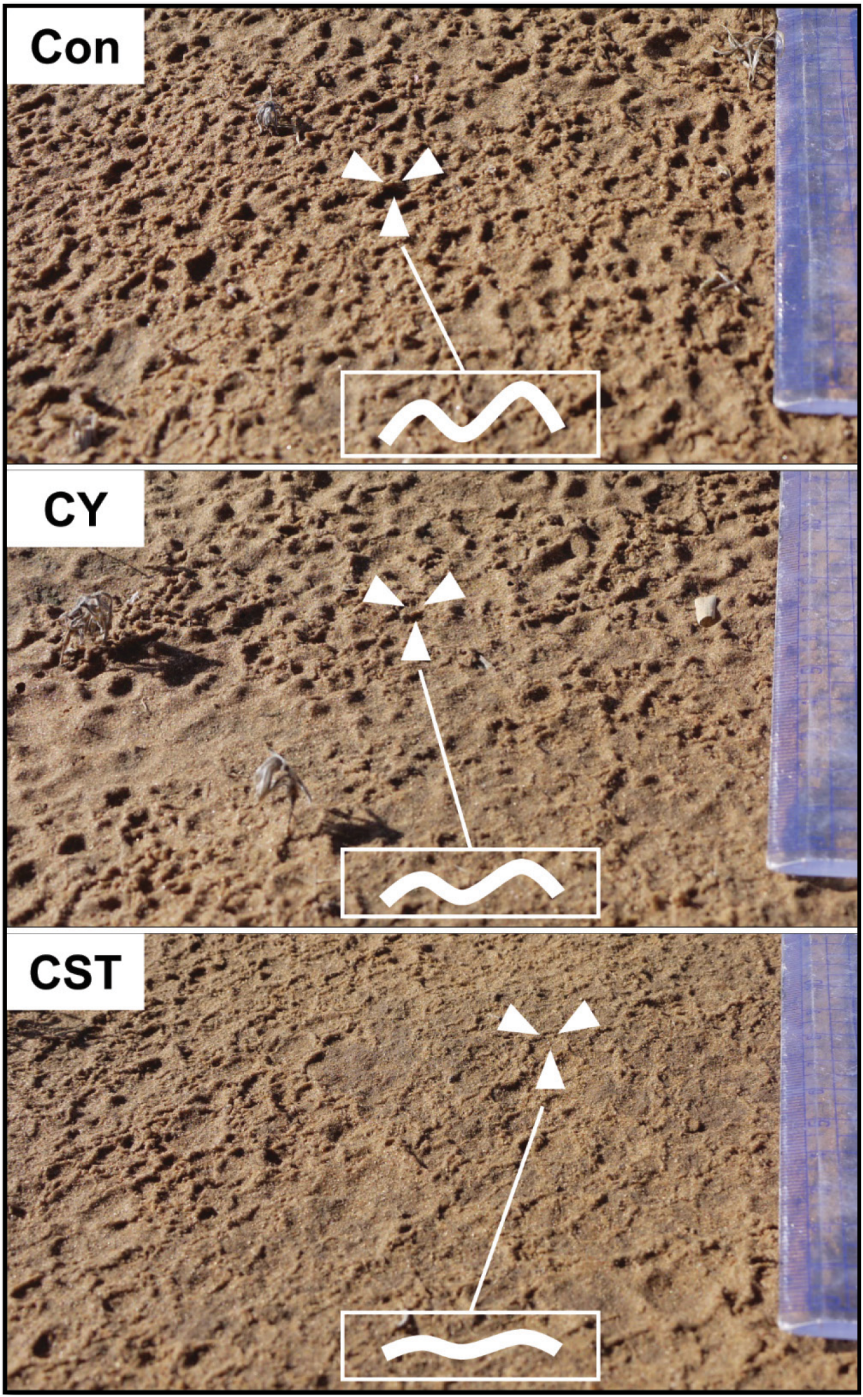
Soil surface of the inoculated crusts immediately after heavy rain in the field. (Con) control soil; (CY) cyanobacteria treated soil; (CST) cyanobacteria with SAP and TKS7 treated soil.

Soil aggregate stability is closely related to EPSs secreted by microorganisms, especially cyanobacteria [47]. In this study, our measurements of cyanobacterial biomass and soil aggregate stability (MWD) increased in proportion to total carbohydrate contents in the inoculated and natural crusts. The CST showed better performance of soil aggregation than CY in the field as well. In addition to polysaccharides secreted from cyanobacteria, adhesive substances of TKS7 had a significant effect on soil aggregation and positively influenced erodibility during 4 months of development, but also sustained the same level of aggregation during 12 months of development. These effects were more evident immediately after rainfall events. As shown in Fig 9, the raindrop imprints of the area subjected to CST appeared to be flat, but the CY and especially Con were uneven following impact by heavy raindrops. Barger *et al*. [48] reported that raindrop impact is the most prominent soil erosion factor. Moreover, organic matter or nutrient loss is higher in cyanobacterial crust in the early stage of formation than in relatively well developed crust owing to weak soil aggregate stability. The results of the present study indicate that raindrop impact caused breakdown and dispersal of soil aggregates on the soil surface, while soil fixing chemicals effectively reduced soil erosion from the inoculated crusts in the early stage of development by improving soil aggregate stability and surface hardness. Furthermore, CST and CY were strong enough to resist erosion by both water and wind. In our study area, the annual number of days with wind speeds higher than 1730 cm s^-1^ was 11. Moreover, the total period with wind speeds higher than 500 cm s^-1^ was 49 days in one year [12]. For these reasons, wind erosion during the early stage of cyanobacterial crust formation with a threshold friction velocity lower than 500 cm s^-1^, such as occurred in the Con soil, is inevitable. Conversely, CST can be protected or less damaged by gales or more continuous wind erosion under natural environmental conditions. Therefore, application of soil fixing chemicals with cyanobacteria can induce more stable cyanobacterial crust in the early stages of formation, and the resulting crust can accelerate successional development of BSC formation in the field.

Biological activities of the inoculated crust should be considered for further evaluation of crust stability and sustainability. Abundant cyanobacterial biomass enhances fixing of soil particles and strengthens soil structure [49–51]. Organic matter is also an important factor in soil aggregation, and soil organic carbon has been shown to be positively correlated with MWD and biomass. Moreover, soil organic carbon is important for nutrient cycling, retaining soil water [52], binding mineral particles [53], reducing aggregate wettability and structural stabilization of soil [54]. Soil respiration and microbial CO_2_ production rate are closely related to the size of microbial biomass pools and soil organic carbon [55]. To evaluate the biological properties listed above, cyanobacterial biomass, microbial biomass carbon, soil respiration, carbon fixation and fluorescence yield were measured in the inoculated crust relative to mature natural crust. The CST or CY significantly enhanced the biological activities compared with bare sand within a few months (*p*<0.05). Furthermore, during 12 months of development, biological activities of inoculated crust, especially in the CST, remained stable or increased. The viability of cyanobacterial cells in the inoculated crust was confirmed by photosynthetic functions such as quantum yield of PSII and relative electron transport rate. The biological activities of the inoculated crusts were estimated to be almost 100% of those of N-BSC within 12 months, while they were 50% after 4 months of development. The carbon fixation of CST and CY reached a similar level as that of 20-year-old N-BSC within 12 months. These findings indicate that rapidly matured crust can play a critical role in accumulating organic carbon and promote the restoration of degraded soil [56]. Despite the use of TKS7, there were no significant differences among biological activities of CST and CY (*p*>0.05), but the physical properties of CST were stronger than those of CY. Park *et al*. [36] investigated whether combined application of TKS7 with cyanobacteria can increase the physical strength of soil relative to the compressional force of a small animal (≤4.9 MPa) by using 0.5 mg dry weight cm^-2^ of TKS7 and 0.5 mg dry weight cm^-2^ of cyanobacterial cells, while singular treatment of TKS7 and cyanobacterial cells were only 1.4 MPa and 2.3 MPa respectively. Furthermore, in addition to having water holding capacity, SAP contains nutrients. These characteristics of SAP help cyanobacterial growth under dry conditions by maintaining high moisture levels [35, 57]. TKS7 is made with tackifiers and polysaccharides derived from plant seeds. Tackifiers include toxic components that may negatively affect cyanobacterial growth [58, 59]. However, no growth inhibition was found when using with SAP [35, 36]. These results suggest that TKS7 and SAP play complementary roles that improve aggregate stability, physical strength and cyanobacterial cell growth [36]. TKS7 must have made a great contribution to soil aggregate stability in the initial step of combined application; however, large filamentous cyanobacteria and its secreted extra cellular polysaccharides will fix soil particles as time goes while TKS7 is degrading. Therefore, a greater aggregate stability value of later stage inoculated cyanobacterial crusts is meaningful and can be used as an index to evaluate settling of inoculated cyanobacteria and their biological role in soil. These findings ruled out the harmful effects of soil fixing chemicals on cyanobacterial cells and the indigenous microbial community while maintaining structural stabilities.

The straw checkerboard system is a key technique for stabilizing sand particles, accumulating organic matter and nutrients. This system has been applied in large arid areas in China and other countries [60, 61]. However, the straw checkerboard system has little worries in automation of border installation at this moment. Accordingly, this technique is only feasible in countries with low labor costs. Under natural conditions, BSCs develop very slowly without any further treatments. The novel technique of combined application of cyanobacteria with SAP and TKS7 has potential for rapid development of cyanobacterial crust in the field within 12 months. When compared to natural BSCs, cyanobacterial crust treated with SAP and TKS7 can reduce the time required for development and the relative cost for recovery. Furthermore, acceleration of ecological succession or recovery will provide great advantages to solve the global desertification problem.

## Conclusions

Naturally formed cyanobacterial crusts in the early stage of development are vulnerable to many destructive factors including rainfall, wind and wildlife. These factors can cause delay of BSC development in the field. However, the novel technique of combined application of cyanobacteria with soil fixing chemicals presented herein can be applied for inoculated cyanobacterial crust formation while maintaining physical properties and biological activities within a few years. Long term monitoring of these inoculated cyanobacterial crusts is needed to confirm the feasibility for timely combating against desertification.
